# Personalized Assessment of Anxiety and Avoidance in Children and Their Parents—Development and Evaluation of the Anxiety and Avoidance Scale for Children

**DOI:** 10.3389/fpsyg.2021.703784

**Published:** 2021-11-17

**Authors:** Michael W. Lippert, Katharina Sommer, Tabea Flasinski, Verena Pflug, Angela Rölver, Hanna Christiansen, Tina In-Albon, Susanne Knappe, Marcel Romanos, Brunna Tuschen-Caffier, Silvia Schneider

**Affiliations:** ^1^Mental Health Research and Treatment Center, Faculty of Psychology, Ruhr University Bochum, Bochum, Germany; ^2^Department of Child and Adolescent Psychiatry, Psychosomatics and Psychotherapy, University Clinic Münster, Münster, Germany; ^3^Clinical Child and Adolescent Psychology, Philipps University Marburg, Marburg, Germany; ^4^Clinical Child and Adolescent Psychology and Psychotherapy, University of Koblenz-Landau, Landau, Germany; ^5^Institute of Clinical Psychology and Psychotherapy, Technische Universität Dresden, Dresden, Germany; ^6^Department of Child and Adolescent Psychiatry, Psychosomatics and Psychotherapy, University Hospital Würzburg, Würzburg, Germany; ^7^Clinical Psychology and Psychotherapy, University of Freiburg, Freiburg, Germany

**Keywords:** personalized assessment, avoidance, anxiety, children, questionnaires

## Abstract

In treating childhood anxiety disorders, therapists use highly individualized anxiety hierarchies to assess anxiety-eliciting situations and to personalize treatment. In contrast, psychometric assessment of anxiety symptoms in children usually consists of standardized questionnaires, assessing either total anxiety or disorder-specific symptom scores, prioritizing comparability over individual information. To account for interindividual differences, the Anxiety and Avoidance Scale for Children (AVAC) was developed, following a precise, personalized, assessment approach. In responding to the questionnaire, children and parents identify the most anxiety-eliciting situations before starting treatment, and rate them for anxiety and avoidance. Ratings are repeated over the course of treatment. The aim of this study is to introduce the new questionnaire and present first data on psychometric properties. The AVAC was administered to 389 children with separation anxiety disorder (*N* = 148), social anxiety disorder (*N* = 110) or specific phobia (*N* = 131) aged 8 to 16 and their parents, along with other measures of anxiety and psychopathology before and after cognitive behavioral treatment. Results showed adequate to good test-retest reliability. The AVAC items correlated significantly with established anxiety questionnaires, indicating convergent construct validity. Regarding divergent construct validity, the AVAC showed only small correlations with externalizing symptoms, demonstrating its precision in measuring anxiety and avoidance. The questionnaire was also sensitive to change after treatment, with medium to large effects in the reduction of anxiety and avoidance. The present analyses suggest that the new personalized assessment approach with the AVAC is a reliable and valid assessment of individualized anxiety and avoidance, as well as change in those constructs over the course of CBT treatment.

## Introduction

Anxiety disorders (AD) are among the most prevalent mental disorders in children and adolescents (Cartwright-Hatton et al., 2006; Polanczyk et al., 2015), causing substantial distress and impairment for children affected, as well as for their families. Further, childhood anxiety disorders (CAD) are an important developmental risk factor (Seehagen et al., 2014), and can serve as a pacemaker for mental disorders in adulthood (Kossowsky et al., 2013).

Anxiety experience is idiosyncratic and modulated by developmental age and individual learning experiences (Costello et al., 2011). Even within a diagnostic spectrum, cognitions, behavior, and anxiety-eliciting situations may vary greatly, and are age-dependent. This is especially true for the diagnostic category of specific phobia, which includes specific fears of different objects (e.g., dogs), environmental stimuli (e.g., thunderstorms), and situations (e.g., going to the dentist). Interindividual differences are found even within types of specific phobia, as well as within other ADs, such as social anxiety disorder. Although children with the same AD show some similarities in anxiety-eliciting situations, differences can occur regarding the severity of anxiety and/or the content of cognitions in certain situations.

In cognitive behavioral therapy (CBT) treatment of CADs, these interindividual differences are often mapped and addressed using highly individualized anxiety hierarchies to guide and plan exposure sessions (Kendall, 1994; Schneider et al., 2013). Typically, these hierarchies are developed at the beginning of therapy in collaboration with the patient, and then later used to determine the sequence of situations to address in graded exposure therapy. Although within treatment protocols, individual cognitions and anxiety-eliciting situations are essential, individual differences are rather neglected in the psychometric assessment of anxieties in children and adolescents. The most common anxiety questionnaires measure either general anxiety or symptoms of specific anxiety disorders, for example the State Trait Anxiety Inventory, the Revised Children Manifested Anxiety Scale, or the Multidimensional Anxiety Scale for Children (Spielberger and Edwards, 1973; Reynolds and Richmond, 1978; March et al., 1997). Most of these questionnaires offer the possibility of calculating a total anxiety score, as well as separate scores for the different AD, thus highlighting different aspects of anxiety without exploring individual symptoms and behaviors. In a recent review, Etkin et al. (2020) investigated eight of the most widely used self-report questionnaires in the field of CADs, and found good to excellent psychometric properties. Regarding test-retest reliability six questionnaires were rated “good” (test-retest correlations *r* ≥ 0.70 over a period of several months), and two were rated “excellent” (test-retest correlations *r* ≥ 0.70 over 1 year or longer). All eight questionnaires showed good construct validity, with correlations ranging from *r* = 0.61 to *r* = 0.81 for convergent validity, and from *r* = 0.07 to *r* = 0.17 for divergent validity. For treatment sensitivity, all questionnaires showed excellent to good quality, in that they were sensitive to change in multiple independent treatment studies. Of these eight questionnaires, only the SCAS (Spence, 1998) uses an item on which patients could provide an individualized answer (“Is there something else that you are really afraid of? Please write down what it is”).

In addition to these, there are also disorder specific questionnaires, which refer to a single category of fears, such as the Social Phobia and Anxiety Inventory, for social anxiety disorder (Beidel et al., 1995), or the Penn State Worry Questionnaire, for generalized anxiety disorders (Chorpita et al., 1997). But even these questionnaires focus on a broad range of frequent cognitions or situations, without the use of individualizable items. In summary, although these questionnaires can be very useful in comparing and classifying patients on a disorder spectrum, they do not account for specific, interindividual differences needed for individualized treatment implementation and evaluation. Therefore, some individual information is lost in favor of standardization and comparability. In addition, most standardized questionnaires are time-consuming, and therefore not appropriate for therapy process research, where short measurements are needed to map changes from session to session.

A personalized assessment of anxiety symptomatology, however, time-consuming, is offered by behavioral assessments such as the behavioral-approach or -avoidance tests (BATs) (e.g., Lester et al., 2011). In these assessments, the approach of a feared object is divided into an individual number of steps. Anxiety and avoidance behavior are measured based on the patient’s ability to master the individual steps. Though highly individualized, this type of test always implies an encounter with the feared object or situation and may therefore be stressful when carried out before treatment. Moreover, when used to monitor therapy progress it is complex, especially in children and adolescents, in that it requires weekly assessment. In addition, providing all the stimuli for individualized assessments (e.g., spiders, dogs, snakes, height, thunderstorms etc.) can be expensive and/or difficult to realize, especially if the focus is on an assessment of treatment progress. Hence, BATs are reliable and usable instruments for organized research projects to measure pre- and post-treatment effects, but are usually too complex to use in session-by-session monitoring of treatment effects or in daily life treatment.

One questionnaire used to individually assess the primary problems to be addressed in treatment is the client-based assessment (Weisz et al., 2004, 2011). In this assessment, the patient’s challenges (behaviors or thoughts) are directly recorded and written down as items in a short questionnaire, with the questionnaire administered regularly as an accompaniment to therapy (Weisz et al., 2011). Weisz et al. (2011) showed that the client-based problem assessment was a reliable (test-retest reliability) and valid (convergent and divergent) instrument that showed sensitivity for change and complemented standardized questionnaires.

To combine the high usability of short questionnaire measures and the more individualized approach of BATs and client-based assessment, the Anxiety and Avoidance Assessment for Children (AVAC) was developed. The AVAC aims to assess individual anxiety-provoking situations, in an economic way, to allow monitoring effects of CBT for CADs throughout the course of treatment following a precise, personalized assessment approach. The child and the parent each report three of the most anxiety provoking situations, as well as each situation’s associated avoidance behavior. For each of these three individualized situations, the severity of anxiety, as well as the associated severity of avoidance behavior, is assessed. To account for consistencies and differences in the perception of anxiety and impairment, both a parent and a child self-report version were developed.

The aim of the present study is to develop a questionnaire for children and adolescents that economically, reliably, and validly measures anxiety and avoidance across a customized set of anxiety-provoking situations. In the first step, a child- and parent-version of the questionnaire was developed. In the second step, the psychometric properties of the AVAC questionnaire were tested in a clinical sample within the framework of a randomized control trial.

First, we assume that regarding reliability, test-retest reliability of the AVAC from two different baseline measurements (baseline 1 and baseline 2) shows satisfactory results. Second, in order to demonstrate construct validity, anxiety and avoidance items of the AVAC are expected to correlate positively with the Spence Children Anxiety Scale (SCAS) as an established anxiety questionnaire, with the Bochum Avoidance and Emotion Regulation Questionnaire for Children (BAER-C) as a measurement of avoidance, and with the subscale of the Strength and Difficulties Questionnaire (SDQ) measuring internalizing symptoms (convergent validity). The correlations with the SCAS are expected to be substantially higher when correlating the AVAC with the separation anxiety or social anxiety subscales in children with a primary diagnosis of either separation anxiety disorder or social anxiety disorder. Third, both AVAC scales should additionally show only a small or no correlation with externalizing symptoms measured by the SDQ scale for externalizing symptoms (divergent validity). Finally, regarding criterion validity (measured by sensitivity of change), the AVAC should show significant differences between baseline and post assessments following CBT treatment for anxiety.

## Materials and Methods

### Participants

Participants included 389 children and adolescents (age *M* = 10.76 years, *SD* = 2.21; range 7–17; 58.2% female), and their parents, who participated in a large randomized controlled trial (KibA therapy study/PEACH trial, GermanCTR ID DRKS00009709) at one of six outpatient clinics for children and adolescents at German universities (Bochum, Marburg, Landau, Freiburg, Dresden and Würzburg). For parent data, only mother’s data were used due to missing data in father’s assessments. Participating families were randomized into one of two CBT treatment conditions, either with or without parental involvement. The study included children with a primary DSM-5 AD, diagnosed with the Diagnostic Interview for Mental Disorders in Children and Adolescents (Kinder-DIPS OA, Margraf et al., 2017; Schneider et al., 2017). Of all children, 37.8% had a primary diagnosis of separation anxiety disorder, 28.2% of social anxiety disorder, and 33.8% of specific phobia. Comorbid anxiety disorder diagnosis was common in all three primary diagnoses. 54.70% of the children with separation disorder suffered from at least one comorbid specific phobia and 14.91% from social anxiety disorder. Only 4.53% had a comorbid externalizing disorder. In children with a primary diagnosis of social anxiety disorder, 44.14% had at least one comorbid specific phobia, while 6.31% suffered from comorbid separation anxiety disorder. Only 2.25% had comorbid externalizing disorders. In specific phobia, 50.38% had at least one additional specific phobia, while 10.15% had comorbid separation anxiety disorder and 10.15% had comorbid social anxiety disorder. 4.51% suffered from comorbid externalizing disorders. All parent data stem from participating mothers. 50.8% of the mothers had at least a high school diploma, 26.3% finished secondary school. 75.6% of the patient’s parents were married or in constant relationship, 8.9% were living alone, 7.9% were divorced and 1% were widowed.

### Rational and Development of the Anxiety and Avoidance Assessment for Children

The AVAC questionnaire was developed as a highly individualized, child and parent-based, efficient measure to assess the level of anxiety and avoidance in anxiety-eliciting situations that children with anxiety may encounter. It was designed to map treatment process and success. Assessment with the AVAC involves asking children at the beginning of therapy to select the individual three most anxiety eliciting situations with the help of the therapist. The questionnaire’s instructions directs children to write down the three situations related to the difficulty which they came to therapy that are the most anxiety-eliciting. They are then given examples for typical situations, including specific phobia situations (dogs, spiders, blood and syringes), social anxiety situations (talk in front of others), and separation anxiety situations (sleepover at a friend’s). In addition, therapists, who fill out the questionnaires with the children and parents, evaluate all answers, and allow those that are concrete situations and not represent worries or thoughts that would not be possible to use for exposure practice (e.g., “I worry about war”). The therapists are asked to ensure that the chosen situations fit the patient’s anxiety diagnosis. If children have another, secondary anxiety diagnosis, situations could also belong to the secondary AD. When all the situations are written down, patients are asked to rate these situations on a five-point Likert-scale for anxiety (0 = no anxiety, 1 = mild anxiety, 2 = medium anxiety, 3 = strong anxiety, 4 = very strong anxiety), as well as avoidance (0 = never avoid, 1 = rarely avoid, 2 = sometimes avoid, 3 = often avoid, 4 = very often avoid). Similar to the personalized assessment by Weisz et al. (2011) three situations were chosen as the sufficient number in order to cover a broad range of situations, while at the same time, keeping the questionnaire short and time efficient. These situations are then used for all treatment assessments.

### Procedure

The AVAC was administered to all children and their parents pre-treatment, post-treatment and after each treatment session. At pre-treatment, children filled out the questionnaire together with their therapist at the end of the diagnostic phase. This was done to ensure that children and their parents chose situations that are relevant to the primary or secondary anxiety diagnosis. To further ensure that parents and children chose situations independently, they filled it out separately from one another.

The questionnaire was then given to the families during baseline 1, baseline 2 (4 weeks waiting time), all intermediates during therapy, post and 6-months follow-up assessments. To ensure that the situations stayed the same throughout therapy all questionnaires were prepared with the situations participants filled out at the beginning.

All children and the parents in the parental involvement condition filled out a paper-pencil version of the questionnaire after each session. The Ethics Committee of the Deutsche Gesellschaft für Psychology (DGPs) approved the study. Local ethics committees validated this with confirmatory votes. The study was pre-registered at the German Clinical Trials Register (GermanCTR ID DRKS00009709).

### Measures

#### Diagnostic Interview for Mental Disorders in Children and Adolescents—Open Access (Kinder-DIPS-OA)

All patients were diagnosed with the Kinder-DIPS-OA (Schneider et al., 2017) by certified assessors, who were either certified psychotherapists for children and adolescents, or in training. All assessors were certified in the reliable and valid use of the interview. The Kinder-DIPS-OA is a well validated structured, clinical interview consisting of a children and parents version to assess DSM-5 diagnosis in children and adolescents (interrater reliability, Cohen’s Kappa = 0.85 to 0.95; for an overview of the psychometric properties, see Neuschwander et al., 2013 and Margraf et al., 2017). Each diagnosis is additionally rated dimensionally, with a severity rating (ranging from 0 to 8). Clinicians combined data from the separately conducted children and parent interviews for the final diagnosis.

#### Spence Children’s Anxiety Scale

The SCAS-C, and its parent version the SCAS-P, are widely known and commonly used instruments to assess anxiety in children and adolescents. The children’s questionnaire consists of 44 items (38 anxiety related, 6 positive filler items), while the parent version excludes the filler items. Both versions measure six domains of anxiety (separation anxiety, social anxiety, obsessive-compulsive disorder, panic/agoraphobia, generalized anxiety). Additionally, all items can be summed for a total anxiety score. In many studies, the questionnaire has shown good to excellent psychometric properties (Spence, 1998; Reardon et al., 2019). The KibA study used the German translation of the SCAS-C and SCAS-P (Essau et al., 2002). This version showed very good internal consistency (Cronbach’s α = 0.92), split-half reliability (*r* = 0.90), as well as convergent validity by correlating significantly with other measures of CADs (*r* = 0.85). In the current study, internal consistency of the scale was α = 0.88 for the child and α = 0.87 for the parent version.

#### Bochum Avoidance and Emotion Regulation Scale for Children

The BAER-C (Lippert et al., submitted) assesses self-reported avoidance as an emotion regulation strategy, as well as reappraisal in anxiety situations in children. Hence, it measures adaptive emotion regulation strategies and avoidance on behavioral (behavioral avoidance), social (verbal and social reassurance), and cognitive (suppression) levels. It is based on the Gross’ process model (Gross, 2001) of emotion regulation and assigns avoidance strategies to the process levels of the model. In its validation study, the total scale showed excellent internal consistency (α = 0.91), with subscales ranging from α = *0.70* to 0.91. In addition, the questionnaire correlated with anxiety symptoms showing convergent validity (*r* = 0.20 to 0.38). In the current study, internal consistency of the total scale was α = 0.89 with subscales ranging from α = *0.71* to 0.90.

#### Strength and Difficulties Questionnaire

The SDQ (Goodman, 2001) is an established screening instrument for psychopathology in children and adolescents. It consists of five subscales (emotional symptoms, conduct problems, hyperactivity/inattention, peer relationship problems, prosocial behavior) and a total of 25 items. The five subscales can be summarized into an externalizing (hyperactivity/inattention and conduct problems), an internalizing (emotional problems, peer relationship problems), and a total score (all four difficulty scales). The child version as well as the parent version showed good to excellent psychometric properties (Cronbach’s α = 0.73; Goodman, 2001), as well as good screening qualities for mental disorders (Goodman et al., 2000), especially for externalizing disorders. In this study, the German self-report and parent report version of the SDQ were used (Lohbeck et al., 2015). In its validation study, the questionnaire showed acceptable to good reliability (Cronbach’s α = 0.55 to 0.77). In the current sample, internal consistency was also good to acceptable for the child (Cronbach’s α = 0.71 for internalizing and α = 0.71 for externalizing scale), as well as the parent version (Cronbach’s α = 0.79 for internalizing and α = 0.78 for externalizing scale).

### Statistical Analysis

Reliability and validity of the AVAC were tested. Due to the personalized diagnostic approach, analyses of internal consistency were not considered, as this is not reasonable. To test for test-retest-reliability, as well as convergent and divergent construct validity, Pearson correlation analyses were conducted using baseline 1 and baseline 2 data. Sensitivity for change was calculated with a series of paired *t*-tests comparing baseline 1 and post-treatment data, corrected with Bonferroni for alpha inflation. All analyses were conducted using IBM SPSS Statistics 24 (IBM Corp, 2016).

## Results

### Test-Retest-Reliability

Test-retest reliability was calculated using data from baseline 1 and baseline 2 assessments. In both the child and mother version, all items correlated significantly between both timepoints (see Table 1).

**TABLE 1:** Test-retest reliability (Pearson correlations) of child and mother-version of the AVAC.

	**Test-retest r**	
	**Anxiety**	**Avoidance**
**AVAC—child**		
Situation 1	0.64**	0.57**
Situation 2	0.54**	0.51**
Situation 3	0.61**	0.56**
**AVAC—mother**		
Situation 1	0.47**	0.74**
Situation 2	0.44**	0.58**
Situation 3	0.50**	0.56**

** p<.001.

### Convergent Validity

#### Child Version

To analyze the AVAC’s convergent and divergent validity, Pearson correlation analyses were conducted. To emphasize the disorder-specific and highly individualized character of the AVAC, results from children with a primary diagnosis of separation anxiety disorder or social anxiety disorder were correlated with the separation anxiety and social anxiety subscales of the SCAS. The correlations were highly significant, therefore supporting construct validity for the anxiety items (all *r* > 0.30, all *p* < 0.001, see Table 2). All three items assessing anxiety on the child version also correlated significantly with the total score of the SCAS-C questionnaire (all *r* > 0.26, *p* < 0.001, see Table 2), as well as with the internalizing subscale of the SDQ self-report (all *r* > 0.18, *p* < 0.001, see Table 2), though these correlations seemed lower than the disorder specific associations. Avoidance, as measured with the AVAC, correlated most strongly with behavioral avoidance in the BAER-C (all *r* > 0.25, *p* < 0.001, see Table 2), thus confirming convergent validity for the assessment of avoidance. In a cross-informant comparison with the parent version of the SCAS, only item two and three of the AVAC reached significance (see Table 2).

**TABLE 2:** Correlations between the AVAC child version and other measures of anxiety symptoms and avoidance (convergent validity) as well as externalizing symptoms (divergent validity).

	**AVAC Child Version**					
	**Anxiety**			**Avoidance**		
	**1**	**2**	**3**	**1**	**2**	**3**
**Anxiety**						
SCAS-C, total	0.28**	0.26**	0.31**	0.08	0.19**	0.19**
SepA Scale^1^	0.52**	0.47**	0.39**	0.21**	0.26**	0.21**
SAD Scale^2^	0.30**	0.35**	0.39**	0.10	0.28**	0.21*
SCAS-P	0.06	0.12*	0.15**	0.03	0.09	0.07
SDQ-self-report-intern	0.18**	0.19**	0.28**	0.10	0.13*	0.11*
SDQ-parent-report-intern	–0.01	0.08	0.11*	0.05	0.13*	0.05
**Avoidance**						
BAER-C—AS	0.23**	0.06	0.08	0.18**	0.11	0.18**
BAER-C—BA	0.32**	0.14*	0.18**	0.32**	0.25**	0.26**
**Externalizing symptoms**						
SDQ-self-report extern	0.08	0.11*	0.13*	0.05	0.04	0.05
SDQ-parent-report-extern	–0.02	0.00	0.03	0.02	0.03	0.09

*^1^Analyzing only children with a primary diagnosis of Separation anxiety disorder (*n* = 148).*

^2^analyzing only children with a primary diagnosis of social anxiety disorder (*n* = 109); AVAC, Anxiety and Avoidance Assessment for Children; SCAS, Spence Child Anxiety Scale; SDQ, Strength and Difficulties Questionnaire (internalizing subscale = intern/externalizing subscale = extern); BAER-C, Bochum Avoidance and Emotion Regulation Scale for Children; AS, Avoidance Score; BA, Behavioral Avoidance; C-Children report; M: Mother report; SepA, Separation Anxiety Disorder; SAD, Social Anxiety Disorder; *p < 0.05; **p < 0.001.

#### Parent Version

The parent version of the AVAC showed similar results. The separation anxiety and social anxiety subscales of the SCAS correlated significantly, when exclusively analyzing data of those children with a primary diagnosis of separation anxiety disorder or social anxiety disorder (all *r* > 0.17, all *p* < 0.001, Table 3). All three situations correlated significantly with total anxiety symptoms *via* parent-report (all *r* > 0.11, all *p* < 0.001, Table 3). Situations two and three showed significant correlations with internalizing symptoms of the SDQ (Table 3). Hence, these results confirmed convergent validity. In contrast to the children’s version, avoidance ratings correlated significantly with anxiety in situation two, whereas correlation with the behavioral avoidance score of the BAER-C were exclusively significant for situation one and three. Cross-informant correlations could only be observed for situation two and partly for situation three (Table 3).

**TABLE 3:** Correlations between the AVAC parent version and measures of anxiety symptoms and avoidance (convergent validity), as well as externalizing symptoms (divergent validity).

	**AVAC Parent Version (mothers)**					
	**Anxiety**			**Avoidance**		
	**1**	**2**	**3**	**1**	**2**	**3**
**Anxiety**						
SCAS-M, total	0.11*	0.24**	0.24**	0.03	0.14**	0.07
SepA Scale^1^	0.21*	0.17*	0.29*	–0.00	0.17*	0.13
SAD Scale^2^	0.28**	0.37**	0.21**	0.16	0.15	0.11
SCAS-C	0.08	0.17**	0.13**	–0.01	0.02	0.10
SDQ-self-report-intern	0.06	0.18**	0.10	–0.03	–0.04	–0.03
SDQ-parent-report-intern	0.09	0.25**	0.18**	0.03	0.07	–0.01
**Avoidance**						
BAER-C—AS	–0.01	0.00	0.11	0.13*	–0.04	0.07
BAER-C—BA	0.07	0.08	0.20**	0.16*	–0.02	0.15*
**Externalizing symptoms**						
SDQ-self-report- Extern	0.04	0.07	0.05	0.05	0.07	0.06
SDQ-parent-report-Extern	0.03	0.16**	0.10	0.10	0.16**	–0.00

^1^Analyzing only children with a primary diagnosis of separation anxiety disorder (*n* = 148). ^2^analyzing only children with a primary diagnosis of social anxiety disorder (*n* = 109); AVAC, Anxiety and Avoidance Assessment for Children; SCAS, Spence Child Anxiety Scale; SDQ, Strength and Difficulties Questionnaire (internalizing subscale = intern/externalizing subscale = extern); BAER-C, Bochum Avoidance and Emotion Regulation Scale for Children; AS, Avoidance Score; BA, Behavioral Avoidance; C, Child Report; M, Mother Report; SepA, Separation Anxiety Disorder; SAD, Social Anxiety Disorder; *p < 0.05; **p < 0.001.

### Divergent Validity

To examine divergent validity, the AVAC was correlated with the externalizing subscale of the SDQ. In children, the anxiety rating of the first situation did not correlate with externalizing symptoms, whereas situation two and three showed small, but significant correlations (Table 2). Anxiety did not correlate with externalizing symptoms rated by parents. Avoidance did not correlate with externalizing symptoms, regardless of the informant (Table 2).

In the parent version, anxiety and avoidance of the AVAC showed significant correlations with externalizing symptoms only in situation two (*r* = 0.16, *p* < *0.001*, Table 3), whereas all other situations showed no significant correlations, thus confirming divergent validity, regardless of the informant.

### Sensitivity to Treatment Change

To examine the AVAC on sensitivity to treatment change, a series of paired *t*-tests were calculated to compare data from baseline 1 and post treatment assessments (Table 4). Patients showed significant improvement in anxiety and avoidance in all three situations after CBT treatment. Cohen’s *d* effect sizes for change in anxiety and avoidance were large for children and parent ratings. In comparison with SCAS-C and SCAS-P, effects for the AVAC were higher (Table 4).

**TABLE 4:** Comparison of Mean AVAC Anxiety, Avoidance, SCAS-C and SCAS-P Scores pre- and post-treatment (sensitivity to change).

**Scale**	**Pre-therapy**	**Post-therapy**	***t*-value**	**Cohen’s**
	***M* (*SD*)**	***M* (*SD*)**	**(df)**	***d***
**AVAC-child anxiety**				
1	3.16 (0.84)	1.52 (1.30)	21.47 (339) **	1.16
2	2.83 (0.97)	1.31 (1.24)	20.49 (326) **	1.13
3	2.65 (1.10)	1.28 (1.21)	18.60 (304) **	1.07
**AVAC child avoidance**				
1	2.83 (1.21)	1.38 (1.42)	16.86 (339) **	0.91
2	2.67 (1.25)	1.36 (1.41)	15.13 (326) **	0.84
3	2.54 (1.34)	1.34 (1.38)	13.93 (303) **	0.80
SCAS-C	28.37 (14.24)	18.06 (12.91)	15.01 (339) **	0.84
**AVAC-parent anxiety**				
1	3.51 (0.68)	1.66 (1.21)	26.74 (319) **	1.50
2	3.25 (0.77)	1.60 (1.25)	22.79 (304) **	1.31
3	3.15 (0.81)	1.52 (1.13)	22.14 (282) **	1.32
**AVAC parent avoidance**				
1	3.16 (1.09)	1.53 (1.28)	21.46 (319) **	1.20
2	3.14 (0.99)	1.51 (1.35)	19.88 (307) **	1.13
3	3.12 (0.92)	1.52 (1.28)	19.92 (283) **	1.18
SCAS-P	30.72 (12.56)	19.38 (10.16)	19.69 (317) **	1.10

AVAC, Anxiety and Avoidance Assessment for Children; SCAS-C, Spence Children’s Anxiety Scale—Children Report; SCAS-P, Spence Children’s Anxiety Scale—Parent Report, **p < 0.001, Bonferroni-corrected.

## Discussion

The aim of the current study was to develop and validate a personalized measure of anxiety and avoidance in the most important anxiety eliciting situations for most CADs. Both parent and children results show that the short assessment is as reliable and valid as classical anxiety questionnaires.

Especially regarding divergent construct validity, the results emphasize the strength of the new questionnaire. Both parent and child data indicate either no significant or a slightly significant correlation between the anxiety/avoidance items and externalizing symptoms measured by SDQ. The AVAC shows lower correlations than the other anxiety questionnaires: SCAS and BAER-C (Essau et al., 2002; Etkin et al., 2020; Lippert et al., submitted), in which anxiety or avoidance scores correlate moderately with externalizing symptoms indicating very good divergent validity. Nevertheless, further studies are needed, especially to investigate the distinction between ADHD and Conduct Disorder, which will complement the present findings on divergent validity.

Regarding convergent construct validity, the AVAC is in line with other anxiety questionnaires. Anxiety and avoidance ratings of the AVAC show significant correlations with anxiety symptoms and behavioral avoidance measured with the BAER-C. Findings are substantially stronger when analyzing the correlations of the disorder-specific SCAS subscales in children with corresponding primary diagnoses (separation anxiety or social anxiety disorder), demonstrating the convergent validity of the AVAC assessment. As expected, the correlations are lower when comparing the AVAC with total anxiety scores of the SCAS. Although these correlations are on the lower end of the range reported by Etkin et al. (2020), they are comparable with similar correlations in studies, which examine other overarching measures of anxiety symptoms with disorder specific assessments (e.g., Liebowotz Social Anxiety Scale for Children and Adolescents and State-trait anxiety inventory for children; Schmits et al., 2014). Unfortunately, there is no subscale or questionnaire included to measure specific phobia symptoms. In future studies the AVAC could close this gap. The correlations of the AVAC avoidance ratings with anxiety symptoms (SCAS and SDQ internalizing) are substantially lower than expected given the theoretical association of these variables. Especially in parents, all correlations except some BAER-C variables are non-significant. This could partly be explained, when examining the rational of the two questionnaires used to measure anxiety and internalizing symptoms. The SCAS aims to identifying the strength of anxiety in different situation, while the SDQ screens for internalizing symptoms in total. Both questionnaires do not assess the behavioral aspect of anxiety, e.g., avoidance behavior. Regarding the parent avoidance correlations, it is possible that parents rate avoidance differently than their children. In addition, some cross-informant correlations were also not significant. Other studies have shown a poor, but usually significant fit between child and parent anxiety rating (Miller et al., 2014). It could be hypothesized that the personal assessment approach of the AVAC allows to depict individual perception of anxiety and avoidance, leading to these low correlations.

Nonetheless, more research with different informants and questionnaires including different aspects of anxiety or even avoidance ratings of structured interviews is necessary to further clarify the AVACs potential to measure avoidance. This should especially target parent ratings to clarify why parent reported avoidance does not correlate with anxiety symptoms. Preliminary, we may assume that parents and children also differ in regard to the behaviors that may serve as avoidance behavior. Reliability analyses show that the individual assessment is stable, by showing good to acceptable test-retest reliability, at a level comparable to the values of the SCAS subscales. However, the correlations are slightly lower than test-retest reliability of the total SCAS score (Ishikawa et al., 2009; Arendt et al., 2014). Because the AVAC measures disorder specific content, the values of the subscales might be better suited for comparison. In addition, the two timepoints used to measure test-retest reliability might have influenced the result as well. While test-retest reliability in the SCAS was usually tested in community samples (Spence, 1998; Zhao et al., 2012), the AVAC was administered before starting treatment. The first assessment was conducted after families finished the diagnostic assessment and gave informed consent for the treatment study. The second assessment was conducted four to 6 weeks later, directly before treatment started. It is possible that the hope of finally starting treatment might have led to a mild improvement in anxiety symptoms. This is in line with research showing that up to 35% of patients waiting for therapy improve slightly but significantly during waiting time for therapy (Young, 2006; Swift et al., 2012). Thus, the AVAC seems to be as valid and reliable as other instruments measuring anxiety and avoidance in children and adolescents.

Lastly, the AVAC’s sensitivity for treatment change was analyzed by comparing pre- and post-treatment scores after CBT treatment. Results show that the AVAC demonstrated large effects between pre- and post-treatment assessment. Effects were stronger for anxiety than for avoidance, and were especially strong when assessed by parents. The effect sizes of the AVAC were larger than effects indicated in meta-analytic studies of treatment effects using the most common, general, overarching anxiety questionnaires (In-Albon and Schneider, 2007; James et al., 2013). The large effect sizes of the AVAC are, however, in line with pre-and post-treatment results from other disorder specific questionnaires, such as the Separation Anxiety Avoidance Inventory (In-Albon et al., 2013). Therefore, future studies might consider including more disorder specific questionnaires, as treatment effects on disorder specific symptoms might be underestimated when using only more general anxiety questionnaires.

In sum, the newly developed individualized AVAC assessment appears to be reliable, valid, and highly sensitive for treatment change, while being a lot less time-consuming than traditional standardized questionnaires. Especially in the context of specific phobia, in which disorder specific questionnaires are scarce, the AVAC could be a valuable addition. Thus, the AVAC is very well suited to be used to monitor the change of anxiety and avoidance throughout the course of treatment for adolescents and for children. The short and individual format of the questionnaire makes it very accessible for children, although some might need more support in the first assessment in writing down the three most important anxiety eliciting situations. Once acquired, the information won by using a client-based assessment can be used to enhance treatment (e.g., exposure). Therapists could plan exposure therapy using the situations described by the patient in the questionnaire and thus adapt exposure therapy to the patient’s needs. This would create an ideal interactive concept for therapy, termed by Weisz et al. (2004) the “assessment-intervention-dialectic,” in which assessments are directly used to plan and individualize treatment. In addition, therapists could use the situations described by the patients to identify anxiety related thoughts and expectations which play an important role in maximizing success of exposure treatment (Craske et al., 2014; Pittig et al., 2016). The AVAC thus provides an important tool in support of therapy, and is innovative in the field of anxiety measures. Our study shows that such a client-based assessment approach can work as well for assessing situations eliciting anxiety and avoidance, as the more general client-based assessments of main problems do (Weisz et al., 2011).

Although the AVAC has shown to be a reliable and valid addition to standardized questionnaires, the client-based assessment format has some limitations. In contrast to standardized questionnaires, children and parents need more support and guidance to fill out the questionnaire in the first assessment. When left unsupervised, children and parents might list situations that do not relate to their primary anxiety diagnosis (e.g., some animals which they might be afraid of sub-clinically. The therapists in the present study were therefore trained to assist the families in writing down the three situations without being suggestive. Similar to the procedure used by Weisz et al. (2011), families filled out the questionnaire directly after they received feedback on their diagnosis to further set their focus on the primary AD. This makes the questionnaire more complex and time-consuming to use for the first time than completely standardized questionnaires. The number of three situations was chosen to keep the questionnaire time efficient, especially when the AVAC is administered for each session. By limiting the number of situations, we aimed to balance between information collection and burden of the informant. However, some information might not have been collected. In addition, a weakness of client-based assessment is its lower comparability, especially across different informants. Because children and their parents are assessed individually, they might choose different situations to be the most important situations, which cannot be compared. This makes it difficult to calculate and compare total scores for the questionnaire. However, this highlights different views and perception of anxiety and visualizes what is most important to the families, which could lead to an increase in motivation and commitment (Weisz et al., 2011). A qualitative analyses and comparison of the content of child and parent assessment is currently under way. Thus, the AVAC is not a replacement of completely standardized questionnaires but a complement, which adds highly individualized information to use in research and especially in treatment of CADs. Finally, treatment effects might be underestimated due to the individual items on the AVAC. When the therapist starts choosing situations the child does not describe in the questionnaire, ratings might not improve, despite the achievements the child makes in treatment. However, this is a limitation which also concerns other questionnaires, and not solely the AVAC.

Future research should explore the AVACs ability to show long term effects of treatment, especially in comparison with standardized questionnaires. It would also be interesting to closely examine the AVAC throughout treatment to investigate treatment process by showing the specific effects of psychoeducation, exposure practice and relapse prevention on anxiety symptoms and avoidance. Another interesting aspect to examine are disorder-specific differences, especially in effect sizes pre to post therapy. These will be part of the treatment study outcome paper. To broaden the understanding of differences in parent-child perception of anxiety eliciting situations, further research could also investigate children’s ratings of parent reported situations and vice-versa. Finally, future studies should make use of the AVAC in the treatment of other ADs, as well as in therapy practice to monitor treatment progress.

In conclusion, the AVAC is a reliable and valid psychometric instrument which complements traditional anxiety questionnaires with a personalized, individual perspective to assess anxiety eliciting situations in children, adolescents, and their parents. In research the questionnaire could further help to understand individual differences in anxiety symptoms even within the same diagnostic spectrum. Its accessibility and shortness make the AVAC ideal to monitor progress over the course of treatment and help to optimize the treatment of CADs.

## Data Availability Statement

The datasets presented in this article are not readily available because data usage will be decided by PROTECT-AD project committee. Requests to access the datasets should be directed to SS, silvia.schneider@rub.de.

## Ethics Statement

The studies involving human participants were reviewed and approved by Ethics Committee of the Deutsche Gesellschaft für Psychologie (DGPs). Written informed consent to participate in this study was provided by the participants’ legal guardian/next of kin.

## Author Contributions

SS, AR, VP, and ML contributed to the conception and design of the questionnaire and the study. ML performed the statistical analysis. ML and KS wrote the first draft of the manuscript. ML, KS, and TF extensively edited the manuscript. SS supervised the writing of the manuscript. All authors contributed to manuscript revision, read, and approved the submitted version.

## Conflict of Interest

The authors declare that the research was conducted in the absence of any commercial or financial relationships that could be construed as a potential conflict of interest.

## Publisher’s Note

All claims expressed in this article are solely those of the authors and do not necessarily represent those of their affiliated organizations, or those of the publisher, the editors and the reviewers. Any product that may be evaluated in this article, or claim that may be made by its manufacturer, is not guaranteed or endorsed by the publisher.
